# Synergistic effects of bast fiber seedling film and nano-silicon fertilizer to increase the lodging resistance and yield of rice

**DOI:** 10.1038/s41598-021-92342-5

**Published:** 2021-06-17

**Authors:** Diankai Gong, Xue Zhang, JiPan Yao, Guijin Dai, Guangxing Yu, Qian Zhu, Qi Gao, Wenjing Zheng

**Affiliations:** 1 Liaoning Rice Research Institute, Shenyang, China; 2 Rice Research Institute, Anhui Academy of Agricultural Sciences, Hefei, China; 3 Inner Mongolia Agricultural University, Hohhot, Inner Mongolia China

**Keywords:** Physiology, Plant sciences

## Abstract

The use of bast fiber film can improve rice seedling quality, and nano-silicon fertilizer can increase rice yields. This study aimed to compare the effects of using bast fiber film, nano-silicon fertilizer, and both treatments on rice yield and lodging resistance. A 2-year field experiment was conducted in 2017 and 2018, in Liaoning, China. The experiment comprised a control (no-bast fiber film, no nano-silicon fertilizer; CK), and three treatments: seedlings cultivated with bast film (FM), single nano-silicon fertilization (SF), and bast fiber film seedlings + nano-silicon fertilization (FM + SF). The japonica rice (*Oryza sativa* L.) cultivar Liaojing 371 was used. Compared with the plants in CK, those in the FM treatment showed greater average root diameter, root volume and root dry weight. The SF treatment increased the single stem flexural strength, increased the contents of silicon, lignin, and cellulose in the rice plant stalk, and reduced the lodging index, thereby increasing lodging resistance. The SF treatment resulted in increased leaf chlorophyll content at late growth stage and a higher net photosynthetic rate, which increased plant dry matter accumulation. In the FM + SF treatment, plant growth was enhanced during the whole growth period, which resulted in an increased number of effective panicles and an increased grain yield. The results show that the combination of FM and SF synergistically improves rice lodging resistance and grain yield. This low-cost, high-efficiency system is of great significance for improving the stability and lodging resistance of rice plants, thereby increasing yields.

## Introduction

Bast fiber film is an eco-friendly and biodegradable seedling raising cloth that is made from bast fibers and small amounts of eco-friendly biodegradable pulp^1,2^. Bast fiber film has been widely used in agriculture in China since 2015 because it is inexpensive, improves seedling quality and uniformity, reduces seedling losses from seedling plates, and ensures better mechanical transplanting^3^. Compared with ordinary plastic film, bast fiber film provides a more constant temperature environment for rice seedlings^4,5^. It is also breathable and hygroscopic. Mulching with bast fiber film on the seedling tray increases the oxygen supply to the soil so that oxygen consumed by soil microbial activity is readily compensated^6^. Using bast fiber film to raise rice seedlings can promote root growth and enhance seedling vitality, and the stronger root systems improve seedling quality. After transplantation into the field, the rice seedlings cultivated with bast fiber film rapidly accumulate photosynthetic pigments and produce tillers early, which increases the number of effective panicles^7,8^. In addition, the bast fiber film is degraded into organic matter, which has a soil-fertilizing effect and thus further stimulates the growth and development of transplanted rice^9^. There is a close and direct relationship between the rice root system and the photosynthetic characteristics of aboveground leaves^10^. In rice plants, rapid pigment accumulation and root growth and development facilitate photosynthesis. This leads to the accumulation of photosynthates in the aboveground parts of rice plants at an early stage of growth, so that more resources are available for grain filling.

As a typical silicon-accumulating crop, rice plants contain large amounts of silicon. The total silicon content in rice plants is greater than the sum of the contents of nitrogen, phosphorus, and potassium^10^. Generally, the SiO_2_ content in stems and leaves reaches 10%–20% on a dry weight basis, so rice is a representative siliceous plants^11^. Silicon plays an important role in rice growth, and large amounts of this mineral are required to maintain normal growth and development^12^. The application of silicon fertilizer can increase chlorophyll content and delay leaf senescence in the late growth stage of rice, thus increasing photosynthesis in the functional leaves^13^. This increases the ability of rice plants to assimilate photosynthetic products during the vegetative growth stage, which improves grain filling and grain quality, and increases resistance and yield^14–16^. Silicon fertilizers can also increase the cellulose content in rice plants during the whole growth period, which in turn hardens the stems and enhances resistance to the lodging and certain diseases. Nano-silica is a novel type of nano-scaled silicon fertilizer made from particles of sparingly soluble materials by nanotechnology methods^17^. Small particle-sized nano-fertilizers are more likely to pass through biological barriers, such as plant cell walls, to fully exert their effects^18^. To date, few studies have evaluated the effects of nano-silicon fertilizers on rice plants.

Previous studies on the use of bast fiber film to raise rice seedlings have reported its beneficial effects on the growth and development of rice at the seedling stage and after transplantation^5^. However, it also results in increased plant height, which increases the risk of lodging^19^. At present, little is known about the effect of bast fiber film on rice plants at the later stages of growth and development. The middle to late stages of grain filling are crucially important for rice yield, and the photosynthetic characteristics in the critical growth period of rice and lodging at later stages determine the magnitude of yield^20–23^. Nano-silicon fertilizers can delay senescence and enhance the resistance of rice plants^24^. Therefore, we speculated that the combination of the blast fiber film and nano-silicon fertilizer might synergistically affect both rice lodging resistance and yield. If so, then this would be a low-cost, simple, and effective way to improve rice production.

## Materials and methods

### Plant materials and experimental design

A field experiment was conducted in 2017 and 2018 at the Rice Research Institute in Liaoning Province, Shenyang, Liaoning, China (N 41° 38′ 31.87″, E 123° 18′ 10.45″). This area is in the North Temperate Zone and has a semi-humid continental climate, with an average annual temperature of 8 °C, an average annual precipitation of 660 mm, and an average annual frost-free period of 170 days. The basic physical and chemical properties of the paddy soil (0–20 cm) were as follows: pH 6.87; organic matter content, 25.13 g/kg^−1^; total nitrogen content, 1.34 g/kg^−1^; available nitrogen content, 85.68 mg/kg^−1^; available phosphorus content, 12.74 mg/kg^−1^; available potassium content, 90.99 mg/kg^−1^; and available silicon content, 176.64 mg/kg^−1^.

The conventional *japonica* rice variety Liaojing 371(medium yield, low to medium lodging level) was used. The bast fiber film used in this study was developed by the Institute of Bast Fiber Crops, Chinese Academy of Agricultural Sciences, and the fertilizer was ‘Silicon Boyuan’ micro-nano-silicon fertilizer. The experiment comprised a control (CK; no bast fiber film, no nano-silicon fertilizer) and three treatments: seedlings raised with bast fiber film (FM), a single nano-Silicon fertilization treatment (SF) and bast fiber film + nano-silicon fertilization (FM + SF). Seeds were sown uniformly with an automatic rice planter at 100 g per tray (60 cm × 30 cm) in soil. Ten seedling trays were established for each treatment. The trays were randomly placed in a greenhouse and managed in the same way as in a conventional dry nursery. Seven days before transplanting, an aqueous solution of micro-nano-silicon fertilizer (concentration, 20% w/v; volume, 100 mL per tray) was evenly applied to seedlings in the SF and FM + SF treatments. The seedlings were transplanted at the 3.5-leaf age at a row spacing of 30 cm × 16.7 cm. The planting area of each plot was 100 m^2^. Each treatment had three replicates and the experiment had a randomized block design. Seeds were sown on April 25th, seedlings were transplanted on May 25th, and the mature plants were harvested on October 1st.

### Measurement of root agronomic traits and plant biomass

Samples were taken from the tray one day before transplanting. Ten representative rice seedlings in each plot were sampled to measure root length and root volume using a root scanner and supporting analytical software (WinRHIZO Pro 2016; Regent Instruments Inc., Quebec City, Canada). At maturity, The dry weight of aboveground parts was determined and five representative plants were selected for analysis(The average number of tillers of ten consecutive rice plants is used as a representative standard). The plants were divided into four parts: ear, stem, leaf, and sheath. The parts were dried separately in an oven at 105 °C for 10 min, and then at 80 °C to constant weight.

### Measurement of SPAD value and photosynthetic characteristics

The upper third of the rice flag leaves was used to measure the SPAD value and photosynthetic characteristics on the 15th day after full heading. The SPAD value and photosynthetic characteristics were measured with a SPAD-502 chlorophyll meter (Minolta, Radiometric Instruments Div., Osaka, Japan) and an LI-6400 portable photosynthesis system (LI-COR, Inc., Lincoln, NE, USA), respectively. The photosynthetic characteristics were net photosynthetic rate (A), stomatal conductance (Gs), intercellular carbon dioxide concentration (Ci), and transpiration rate (E). These measurements were conducted between 9 and 11 am on a sunny day.

### Measurement of lodging resistance-related traits

On the tenth days before maturity, rice plants from five hills per plot were selected, five representative tillers with consistent growth were chosen using the secondary sampling method, and the average value was determined. The plant height, center of gravity height, basal internode (including leaf sheath) length, stem diameter, and dry matter weight were recorded. The internode breaking resistance was measured with a stem strength tester (Model: YYD-1,Zhejiang Top Instruments, Zhejiang, China)^25^. The stems were collected from mature plants. The silicon concentration in the plant stem was determined by the colorimetric molybdenum blue method^26^, and cellulose and lignin contents were determined using Van Soest’s washed fiber analysis method^27^.

### Measurement of yield and its components

At harvest, the rice yield was measured for an area of 10 m^2^ per treatment. After drying and threshing, the rice grains were weighed to calculate grain yield. The number of effective panicles in 20 consecutively growing rice plants in each treatment was counted. Use the average number of tillers as the standard for selecting rice to determine the yield components. The number of grains per panicle, seed setting rate, 1000-grain weight and the grain yield were recorded.

### Data analysis

Data were processed and analyzed using GraphPad Prism 8 and SPSS 18 statistical software. Significant differences among treatments were detected using two-way ANOVA.

My experimental research and field studies on *Oryza sativa L* (either cultivated or wild), including the collection of plant material, comply with relevant institutional, national, and international guidelines and legislation.

## Results

### Effects of bast fiber film and nano-silicon fertilizer treatments on rice yield

**Figure 1:**
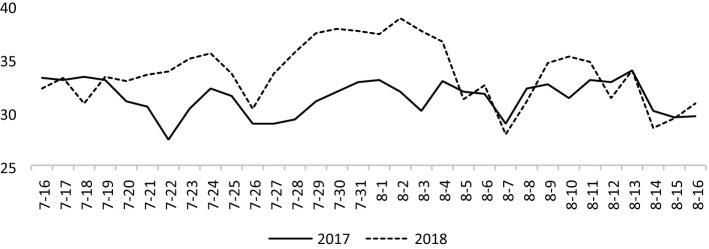
Daily maximum temperature during the flowering and grain filling period of rice plants in 2017 and 2018.

Both the FM and SF treatments had significant positive effects on the number of panicles (Table 1). The number of panicles in 2017 and 2018 was increased by 11.4%–4.5%, respectively, in the FM + SF treatment compared with CK. As shown in Fig. 1, the maximum temperature from July to August in 2018 was higher than that in previous years, which affected the overall rice yield and yield structure.The high temperature during the flowering period in 2018 resulted in a general decrease in grain yield, accompanied by lower panicle number, seed setting rate, and 1000-grain weight, compared with their respective values in 2017. The seed setting rate was significantly among treatments in 2018, and was higher in the FM and FM + SF treatments than in CK. The 1000-grain weight of rice significantly higher (by about 3%) in the SF and FM + SF treatments than in CK. The yield was about 8% higher in the FM + SF treatment than in CK; this difference was statistically significant.

**Table 1:** Effects of bast fiber film and nano-silicon fertilizer on rice yield and yield components.

Year	Treatment	Yield (t/hm^2^)	Panicle number (10,000 /hm^2^)	Grain number per panicle	Seed setting rate (%)	1000-grain weight (g)
2017	CK	9.80 ± 0.44b	377.2 ± 9.9b	105 ± 5a	91.18 ± 0.55a	27.20 ± 0.10b
	FM	10.25 ± 0.23ab	403.9 ± 16.2ab	103 ± 5a	91.89 ± 0.66a	26.83 ± 0.15c
	SF	10.15 ± 0.49ab	393.7 ± 16.5ab	102 ± 5a	91.17 ± 0.25a	27.83 ± 0.06a
	FM + SF	10.62 ± 0.33a	420.2 ± 9.8a	100 ± 6a	92.68 ± 1.31a	27.70 ± 0.10a
2018	CK	8.68 ± 0.10b	331.9 ± 14.2a	101 ± 7a	84.27 ± 0.41c	25.23 ± 0.06b
	FM	9.05 ± 0.13ab	339.9 ± 19.2a	113 ± 6a	90.93 ± 0.78a	26.37 ± 0.06a
	SF	9.22 ± 0.39ab	334.8 ± 14.4a	112 ± 5a	87.58 ± 1.21b	26.47 ± 0.06a
	FM + SF	9.38 ± 0.42a	379.9 ± 39.3a	107 ± 6a	89.37 ± 1.19ab	26.23 ± 0.23a
Y		278.67**	87.26**	3.28 ns	9.10 ns	17.78*
T		21.46*	12.31*	0.53 ns	1.90 ns	1.75 ns
Y*T		0.23 ns	0.48 ns	2.09 ns	11.64**	42.55**

Different letters indicate significant difference at 5% level. Summary of ANOVA results: not significant (ns), significant at *p* < 0.05 (*), very significant at *p* < 0.01 (**).

*Y* Year, *T* Treatment.

### Effects of bast fiber film and silicon fertilizer on rice seedling quality

The root projected area, root surface area, and root volume differed significantly among treatments (Table 2). The 2-year average root length was significantly increased by 11.1% in the FM treatment and by 27.6% in the FM + SF treatment compared with that in CK. The 2-year average values of root projected area, surface area, and root volume were 38.9%, 42.6%, and 54.8% higher, respectively, in the FM + SF treatment than in CK. The average diameter of seedling roots was higher in the FM and SF treatments than in CK in 2018, but not significantly different among the treatments in 2017.

**Table 2:** Effects of bast fiber film and nano-silicon fertilizer treatments on root index of rice at the seedling stage.

Year	Treatment	Root length(cm)	Projected area (cm^2^)	Surface area (cm^2^)	Mean diameter (mm)	Root volume (cm^3^)	Root tip number
2017	CK	94.11 ± 1.94b	2.86 ± 0.33b	8.97 ± 1.04b	0.30 ± 0.03a	0.07 ± 0.01a	693.67 ± 142.94a
	FM	105.34 ± 3.22a	3.68 ± 0.57a	11.57 ± 1.78ab	0.36 ± 0.03a	0.11 ± 0.03a	653.50 ± 140.50a
	SF	96.77 ± 6.20b	3.00 ± 0.31b	10.34 ± 2.08ab	0.34 ± 0.05a	0.09 ± 0.03a	467.75 ± 21.26b
	FM + SF	110.95 ± 2.20a	3.71 ± 0.13a	12.08 ± 0.19a	0.34 ± 0.01a	0.10 ± 0.01a	576.06 ± 150.68ab
2018	CK	95.46 ± 0.91c	2.71 ± 0.26c	8.48 ± 0.82b	0.28 ± 0.03b	0.06 ± 0.01b	628.92 ± 85.67c
	FM	105.34 ± 3.23b	3.68 ± 0.57ab	11.57 ± 1.78a	0.36 ± 0.03a	0.11 ± 0.03a	653.50 ± 140.50b
	SF	99.45 ± 3.05c	3.07 ± 0.21bc	11.28 ± 1.30a	0.35 ± 0.03a	0.10 ± 0.02a	565.75 ± 24.13b
	FM + SF	131.19 ± 3.89a	4.00 ± 0.32a	12.56 ± 1.00a	0.31 ± 0.02ab	0.10 ± 0.01a	871.33 ± 105.46a
Y		1.63 ns	0.34 ns	0.57 ns	1.04 ns	0.12 ns	1.09 ns
T		6.05 ns	31.58**	25.17*	7.29 ns	27.64*	1.23 ns
Y*T		11.65**	0.37 ns	0.30 ns	0.73 ns	0.19 ns	2.90 ns

Different letters indicate significant difference at 5% level. Summary of ANOVA results: not significant (ns), significant at *p* < 0.05 (*), very significant at *p* < 0.01 (**).

*Y* Year, *T* Treatment.

### Effects of bast fiber film and silicon fertilizer treatments on SPAD value, photosynthetic characteristics, and dry matter allocation

The A and Gs values were 19.1% and 16.9% higher, respectively, in the FM + SF treatment than in CK. There were no significant differences in Ci and E among treatments. The SPAD values were significantly higher in FM + SF and SF treatments than in CK. The A, Gs, E, and SPAD values of plants in the various treatments are shown in Table 3.

**Table 3:** Effects of bast fiber film and nano-silicon fertilizer treatments on SPAD and photosynthetic characteristics of rice flag leaves at mid-filling stage.

Year	Treatment	Pn (μmol CO_2_ m^−2^ s^−1^)	Cond (μmol H_2_O m^−2^ s^−1^)	Ci (μmol CO_2_ mol^−1^)	Trmmol (mmol m^−2^ s^−1^)	SPAD
2017	CK	14.28 ± 0.15c	0.59 ± 0.05b	302.20 ± 4.21a	7.03 ± 0.17a	34.9 ± 0.15c
	FM	15.84 ± 0.58ab	0.65 ± 0.03ab	303.60 ± 2.70a	6.97 ± 0.14a	35.1 ± 0.26c
	SF	15.06 ± 0.68bc	0.61 ± 0.04ab	303.60 ± 1.82a	7.19 ± 0.21a	35.8 ± 0.40b
	FM + SF	16.54 ± 1.14a	0.69 ± 0.11a	303.80 ± 6.06a	7.06 ± 0.31a	36.6 ± 0.12a
2018	CK	14.10 ± 0.20c	0.60 ± 0.06a	302.40 ± 3.29a	7.09 ± 0.28a	35.5 ± 0.35c
	FM	15.86 ± 0.57b	0.61 ± 0.08a	302.40 ± 4.16a	7.00 ± 0.16a	36.4 ± 0.20b
	SF	15.30 ± 0.48b	0.62 ± 0.02a	303.60 ± 1.82a	7.22 ± 0.16a	36.7 ± 0.15b
	FM + SF	17.30 ± 0.83a	0.68 ± 0.03a	305.80 ± 7.53a	7.12 ± 0.10a	37.2 ± 0.25a
Y		1.07 ns	0.82 ns	0.14 ns	36.95*	27.22*
T		32.16 **	10.88 *	2.58 ns	159.06*	32.89*
Y*T		0.96 ns	0.37 ns	0.23 ns	0.01 ns	1.88 ns

Different letters indicate significant difference at 5% level. Summary of ANOVA results: not significant (ns), significant at p < 0.05 (*), very significant at p < 0.01 (**).

*Y* Year, *T* Treatment, *Pn* Net photosynthetic rate, *Cond* Stomatal conductance, *Ci* Intercellular carbon dioxide concentration. *Trmmol* Transpiration rate.

As shown in Table 4, the biological yield and the sheath dry weight of rice were significantly higher in the FM treatment than in CK in both 2017 and 2018. The harvest index was 6.5% and 4.2% higher in the FM + SF treatment than in CK in 2017 and 2018, respectively. Because of the influence of temperature during the flowering period in 2017 and 2018, the effects of FM and SF on yield were slightly different between the 2 years, but the trend was the same.

**Table 4:** Effects of bast fiber film and nano-silicon fertilizer treatments on dry matter allocation in rice at mature stage.

Year	Treatment	Biological yield (t ha^−1^)	Sheath dry weight (t ha^−1^)	Stem dry weight (t ha^−1^)	Leaf dry weight (t ha^−1^)	Harvest index
2017	CK	21.17 ± 0.31b	4.44 ± 0.38ab	2.78 ± 0.27ab	4.16 ± 0.24a	0.46 ± 0.01bc
	FM	22.70 ± 0.82a	4.88 ± 0.22a	3.22 ± 0.43a	4.34 ± 0.14a	0.45 ± 0.01c
	SF	20.59 ± 0.51b	4.23 ± 0.31b	2.47 ± 0.39b	3.74 ± 0.56a	0.49 ± 0.02a
	FM + SF	21.71 ± 0.52ab	4.23 ± 0.29b	2.65 ± 0.32ab	4.2 ± 0.24a	0.49 ± 0.02ab
2018	CK	18.03 ± 1.34a	3.72 ± 0.16b	2.39 ± 0.33a	3.24 ± 0.43a	0.48 ± 0.03ab
	FM	19.65 ± 0.47a	4.22 ± 0.12a	2.61 ± 0.2a	3.77 ± 0.19a	0.46 ± 0.00b
	SF	19.33 ± 0.43a	4.1 ± 0.09a	2.48 ± 0.09a	3.54 ± 0.46a	0.48 ± 0.01ab
	FM + SF	18.67 ± 1.29a	3.68 ± 0.23b	2.24 ± 0.25a	3.36 ± 0.46a	0.5 ± 0.02a
Y		254.28*	15.16 ns	7.19 ns	14.37 ns	6.23 ns
T		1.70 ns	4.22*	2.75 ns	1.12*	2.98 ns
Y*T		1.60 ns	0.41 ns	1.12 ns	0.90 ns	1.24 ns

Different letters indicate significant difference at 5% level. Summary of ANOVA results: not significant (ns), significant at p < 0.05 (*), very significant at p < 0.01 (**).

*Y* Year, T Treatment.

### Effects of bast fiber film and silicon fertilizer on lodging resistance of rice at late filling stage

As shown in Table 5, the 2-year average values of plant height and center of gravity were significantly higher in the FM treatment than in CK. The length of the first, second and third sections in the SF treatment were shorter than their corresponding counterparts in CK, while the stem diameter was slightly greater than that of CK. The dry weight per unit length of the first and the second nodes was significantly higher in the FM + SF treatment than in CK.

**Table 5:** Effects of bast fiber film and nano-silicon fertilizer treatments on lodging resistance traits in rice.

Year	Treatment	Plant height (cm)	Center of gravity height (cm)	First node			Second node			Third node		
				Internode length (cm)	Stem diameter (mm)	Unit length dry weight (g/cm)	Internode length (cm)	Stem diameter (mm)	Unit length dry weight (g cm^−1^)	Internode length (cm)	Stem diameter (mm)	Unit length dry weight (g/cm)
2017	CK	118.00b	34.70b	6.36a	5.37b	0.07b	11.71ab	5.03a	0.04b	15.48ab	4.23a	0.04a
	FM	123.60a	35.60a	5.69a	5.61ab	0.06b	12.51a	5.02a	0.03b	17.20a	4.06a	0.02a
	SF	115.70b	33.70b	2.64b	6.06a	0.13a	10.14bc	5.05a	0.04b	14.71b	4.18a	0.03a
	FM + SF	116.70b	34.00b	3.39b	6.06a	0.13a	9.74c	5.10a	0.05a	14.12b	4.40a	0.03a
2018	CK	116.70a	32.90a	5.72a	5.41c	0.07b	11.39a	4.92a	0.04b	15.6ab	4.21a	0.03a
	FM	119.70a	33.30a	5.18b	5.68bc	0.07b	11.78a	4.88a	0.04b	17.00a	4.13a	0.03a
	SF	111.30b	31.50b	2.8c	6.23a	0.11a	10.07b	5.07a	0.05a	14.47bc	4.19a	0.03a
	FM + SF	112.00b	31.80b	3.06c	5.93ab	0.11a	9.65b	5.05a	0.05a	13.02c	4.19a	0.03a
Y		14.92*	286.92**	1.59 ns	0.31 ns	0.97 ns	5.32 ns	2.41 ns	7.76 ns	2.98 ns	0.69 ns	0.49 ns
T		15.27*	45.63**	45.85**	26.87*	17.25*	66.65**	3.18 ns	4.67 ns	34.30**	2.19 ns	1.32 ns
Y*T		2.15 ns	0.39 ns	1.59 ns	0.94 ns	1.15 ns	0.80 ns	0.25 ns	2.77 ns	0.68 ns	1.17 ns	1.44 ns

Different letters indicate significant difference at 5% level. Summary of ANOVA results: not significant (ns), significant at p < 0.05 (*), very significant at p < 0.01 (**).

*Y* Year, *T* Treatment.

As shown in Table 6, the bending moment and lodging index of all nodes were significantly higher in the FM treatment than in CK. The breaking resistance and bending moment of all nodes, except the second and third nodes in 2017, were significantly higher in the SF treatment than in CK, while the lodging index of the first node was lower in the SF treatment than in CK. The FM + SF treatment resulted in increased breaking resistance and decreased lodging index of the first node, relative to those in CK but did not affect any other node parameters.

**Table 6:** Effect of bast fiber film and nano-silicon fertilizer treatments on lodging resistance in rice.

Year	Treatment	First node			Second node			Third node		
		Breaking resistance (N)	Bending moment (cm/g)	Lodging index	Breaking resistance (N)	Bending moment (cm/g)	Lodging index	Breaking resistance (N)	Bending moment (cm/g)	Lodging index
2017	CK	39.05bc	1596.47b	41.70b	24.38a	1231.71b	53.44b	17.65a	992.17a	63.9ab
	FM	33.44c	1792.15a	54.68a	16.53b	1403.96a	86.23a	13.61b	996.09a	74.51a
	SF	60.00a	1627.69b	27.78c	26.25a	1396.66a	54.25b	17.70a	1053.38a	60.13b
	FM + SF	44.83b	1641.98b	37.13b	26.08a	1385.82a	52.03b	17.63a	1058.34a	61.81ab
2018	CK	38.84b	1520.74b	39.28b	21.61b	1251.16b	57.94b	15.66b	945.79c	60.4b
	FM	34.5b	1649.51a	47.95a	19.11c	1387.64a	72.63a	13.75c	1050.44ab	76.44a
	SF	50a	1526.68b	30.57c	23.83a	1380.91a	58.02b	17.63a	1093.23a	61.99b
	FM + SF	45.03a	1418.2c	31.6c	23.21ab	1267.62b	54.62b	15.91b	1006.59bc	63.35b
Y		0.49 ns	2.14 ns	0.11 ns	0.60 ns	2.11 ns	0.02 ns	0.24 ns	1.46 ns	0.07 ns
T		8.76 ns	1745.43**	27.27*	6.36 ns	–**	7.99 ns	20.68*	–**	29.44*
Y*T		12.04**	0.02 ns	4.53*	11.10**	–ns	12.84**	1.37 ns	–ns	1.44 ns

Different letters indicate significant difference at 5% level. Summary of ANOVA results: not significant (ns), significant at p < 0.05 (*), very significant at p < 0.01 (**).

*Y* Year, *T* Treatment.

**Figure 2:**
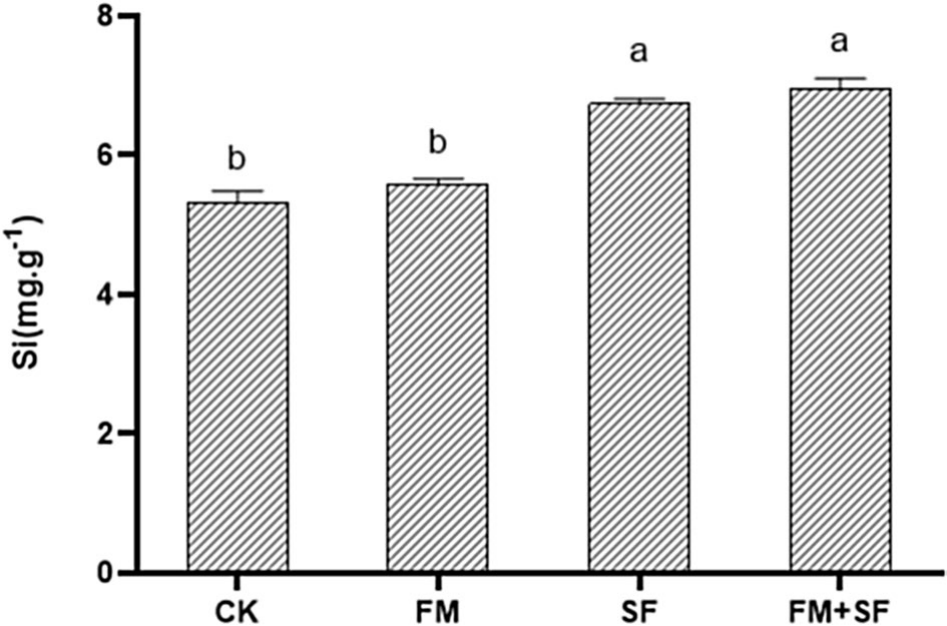
Effect of bast fiber film and nano-silicon fertilizer treatments on silicon content in rice stalks. Different letters indicate significant difference at 5% level. Control (CK), no bast fiber film, no nano-silicon fertilizer; FM, seedlings cultivated with bast film; SF, seedlings cultivated with nano-silicon fertilizer; FM + SF, seedlings cultivated with bast fiber film and nano-silicon fertilizer.

The application of nano-silicon fertilizer significantly increased the silicon content in rice stems. The stem silicon content in the FM + SF and SF treatments was 6.94 mg.g^−1^ and 6.74 mg.g^−1^, respectively, 38% and 34% higher than that in CK (Fig. 2).

**Figure 3:**
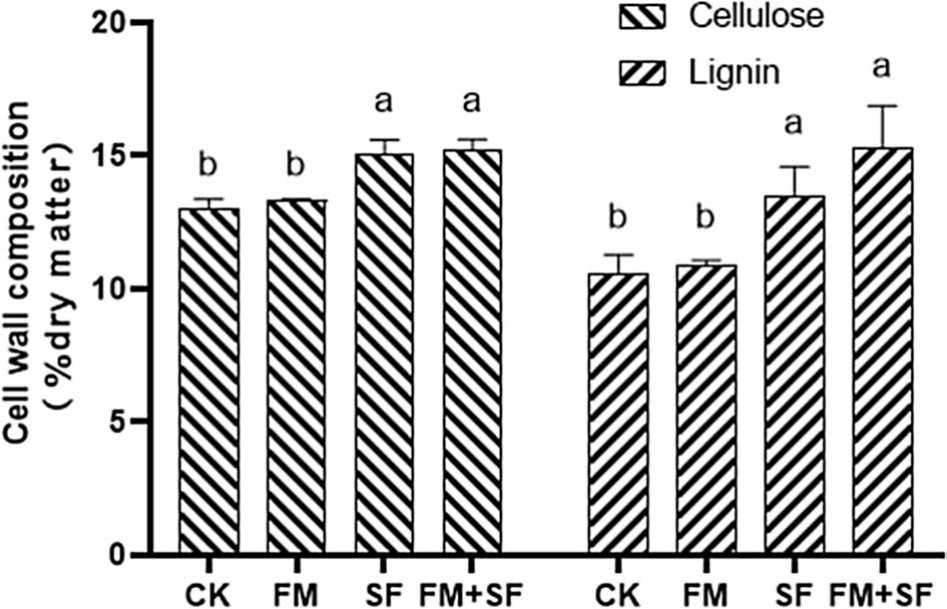
Effect of bast fiber film and nano-silicon fertilizer treatments on cellulose and lignin content in rice stalks. Different letters indicate significant difference at 5% level. Control (CK), no bast fiber film, no nano-silicon fertilizer; FM, seedlings cultivated with bast film; SF, seedlings cultivated with nano-silicon fertilizer; FM + SF, seedlings cultivated with bast fiber film and nano-silicon fertilizer.

The cellulose and lignin contents in stems were also significantly higher in the SF and FM + SF treatments than in CK (Fig. 3). The cellulose content in the stems was 15.21% in the FM + SF treatment, 17% higher than that in CK. The lignin content in the stems was 15.30% in the FM + SF treatment, 44% higher than that in CK. The cellulose and lignin contents in stalks did not differ significantly between FM and CK.

## Discussion

Because bast fiber film is a breathable mulch, oxygen consumed at the bottom of rice seedling discs is readily replaced, similar to the case in aerobic cultivation^28^. The use of bast fiber film benefits the root growth of rice seedlings. It can promote root primordia differentiation, which increases the numbers of adventitious roots and denser and better-quality root networks^29^. Mulching with bast fiber film can also increase rice seedling root vitality and the number of white roots, which contributes to whole plant growth. This leads to improved seedling quality and faster pigment accumulation after transplanting^30^. Higher dry matter accumulation in above-ground parts of seedlings can induce earlier tillering after transplanting, which resulted in an increased number of effective tillers and higher yields^31–33^. The results of this study show that the use of bast fiber film can promote the growth and development of rice seedling roots. In this study, seedlings cultivated with bast fiber film showed increased dry weight, mean diameter, volume, and surface area of roots, increased stem base diameter and aboveground growth, and improved plant quality. These attributes resulted in rapid greening after transplanting and better growth and development of photosynthetic organs. These effects resulted in rapid dry matter accumulation, which provided the basis for higher and more stable rice yields.

The SPAD value is an indirect index of leaf chlorophyll content^34^. In this study, the use of bast fiber film to cultivate seedlings and the application of nano-silicon fertilizer at the seedling stage increased the chlorophyll content of rice leaves and delayed senescence, thereby enhancing photosynthesis in functional leaves. This improved the capacity of rice plants to assimilate photosynthetic products during the growth stage. Our results also show that the use of bast fiber film to cultivate seedlings resulted in increased dry matter accumulation in leaves and stems, thereby increasing biological and grain yields. Using bast fiber film to cultivate seedlings or applying nano-silicon fertilizer at the seedling stage slightly increased yield compared with that of the control, but the combination of these two treatments increased the effective panicle number per unit area, which significantly increases the yield.

In the rice plants in the combined bast fiber film and nano-silicon fertilizer treatment, the flag leaf chlorophyll content at the late growth stage was 4.8% higher, the net photosynthetic rate was 10.9% higher, and the dry weight at the mature stage was 9.5% higher than their respective values in the control. Our results show that the use of bast fiber film can promote seedling regeneration after transplanting and that the application of silicon fertilizer can delay senescence, enhance the photosynthetic capacity, and increase dry matter accumulation, thereby boosting yields.

Liu et al.^35^ reported that a high lignin level in the cell wall improves lodging resistance, and suggested target genes for the genetic modification of lignin content to breed rice lines with high lodging resistance. Our results show that the application of nano-silicon fertilizer can significantly increase the contents of silicon, lignin, and cellulose in stalks, shorten internode length, and reduce plant height. All these changes increase lodging resistance. In addition, the application of nano-silicon fertilizer can significantly increase the dry weight per unit length, which lowers the lodging index and increases lodging resistance.

## Conclusions

Using bast fiber film to cultivate rice seedlings can promote growth at the cost of increasing the risk of lodging. Applying nano-silicon fertilizer can delay senescence and improve the lodging resistance of rice plants. Our results show that the combination of bast fiber film to cultivate rice seedlings and nano-silicon fertilizer synergistically affects rice yield, lodging resistance, root characteristics, and leaf photosynthetic traits. Thus, this is a low-cost and simple method to improve rice yields effectively and efficiently.
